# Portable digital microscopy with point-of-care testing for low-cost and efficient prevalence surveys for schistosomiasis control

**DOI:** 10.1371/journal.pntd.0013444

**Published:** 2025-10-03

**Authors:** Isaac I. Bogoch, Jean T. Coulibaly, Kigbafori D. Silue, Karla N. Fisher, María Díaz de León Derby, Daniel A. Fletcher, Nathan C. Lo

**Affiliations:** 1 Department of Medicine, University of Toronto, Toronto, Canada; 2 Unité de Formation et de Recherche Biosciences, Université Félix Houphouët-Boigny, Abidjan, Côte d’Ivoire; 3 Centre Suisse de Recherches Scientifiques en Côte d’Ivoire, Abidjan, Côte d’Ivoire; 4 Swiss Tropical and Public Health Institute, Basel, Switzerland; 5 University of Basel, Basel, Switzerland; 6 Divisions of General Internal Medicine and Infectious Diseases, Toronto General Hospital, University Health Network, Toronto, Canada; 7 Department of Bioengineering, University of California, Berkeley, Berkeley, California, United States of America; 8 Biological Systems and Engineering Division, Lawrence Berkeley National Laboratory, University of California, Berkeley, Berkeley, California, United States of America; 9 Chan Zuckerberg Biohub, San Francisco, California, United States of America; 10 Division of Infectious Diseases and Geographic Medicine, Department of Medicine, Stanford University, Stanford, California, United States of America; Universidad de la República Uruguay: Universidad de la Republica Uruguay, URUGUAY

Schistosomiasis is a neglected parasitic disease, caused by the trematode *Schistosoma* spp., and is estimated to infect 150–250 million persons in low- and middle-income countries [1,2]. WHO guidelines recommend annual preventive chemotherapy (delivered through mass administration of praziquantel) to all persons 2 years and older in geographic areas endemic with *Schistosoma*, defined by having 10% or greater prevalence of infection by microscopy methods (with alternative thresholds using an alternative diagnostic tool) [3]. WHO has targeted schistosomiasis for elimination as a public health problem by 2030, but many challenges remain, including “hot spots” of persistent endemicity that are less responsive to annual preventive chemotherapy [3–5]. After decades of preventive chemotherapy, endemic areas and hot spots are becoming increasingly geographically focal. Therefore, a key challenge to schistosomiasis control and elimination is performing more precise, cost-efficient, and tailored prevalence surveys to identify endemic and hot spot locations to guide customized preventive chemotherapy [5]. This article explores the potential of portable digital microscopy (for *S. haematobium* infections) to strengthen schistosomiasis diagnosis for prevalence surveys when coupled with a Point-of-Care Circulating Cathodic Antigen (POC-CCA) assay (for *S. mansoni* infections), providing a discussion of their benefits, hypothetical cost estimate, and challenges for their implementation.

Historically, surveys that are designed to estimate *Schistosoma* spp. infection prevalence predominatly rely on microscopy-based diagnostics that are slow, low-sensitivity, and are resource-intensive [3,6,7]. The conventional diagnostic tools rely on light microscopy for detection of eggs; *S. mansoni* endemic areas use stool microscopy with the Kato–Katz technique (varying the number of stool samples and slides examined, sometimes over multiple days), while *S. haematobium* endemic areas perform urine filtration with light microscopy [7]. Newer diagnostics, such as point-of-care rapid urine antigen assays, are a recent advance but only commercially available for *S. mansoni* through the POC-CCA [2,3,6]. While prevalence surveys are commonly conducted on a district level, there is increasing interest in conducting more spatially granular (e.g., sub-district or community) surveys to guide tailored preventive chemotherapy. However, these microscopy-based diagnostic tools pose a challenge to scaling up to map schistosomiasis at a finer spatial scale.

**Fig 1:**
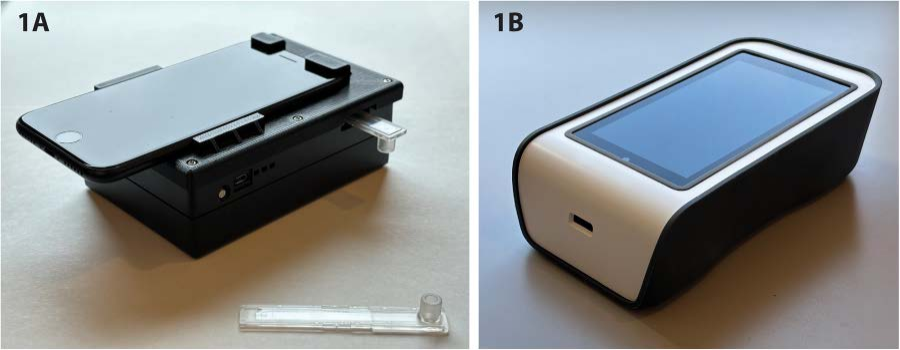
Portable digital microscope tools for schistosomiasis diagnosis. **(A)** Earlier prototype featuring a mobile phone mounted on a 3D-printed base with a capillary tube inserted for sample imaging (image adapted from [10]). **(B)**. Updated version with integrated optics and enhanced functionality, including onboard AI capabilities (image adapted from [11]).

In recent years, portable digital microscopy has emerged as a potential alternative diagnostic tool for public health application for schistosomiasis, offering the possibility of being a low-cost, fast, and accessible tool for conducting prevalence surveys for schistosomiasis (Fig 1) [8–13]. Many of these tools have been developed and used across the globe over the past decade. While tested with many infectious diseases, they have particularly promising data for fast, sensitive, and simple diagnosis of *S. haematobium* [10]. Early versions of portable digital microscopes have used mobile phones mounted on 3D-printed structures to magnify pathogens for manual identification by microscopists (example shown in Fig 1A) [10]. In some cases, newer models that retain similar core components can be produced at scale with integrated optics and electronics (Fig 1B) [11]. These devices use a mechanized stage for scanning capillary tubes containing 10 ml of filtered urine designed to trap *S. haematobium* eggs for visualization. Newer microscopes may integrate artificial intelligence software to enable automated detection and quantification of the eggs [14]. In a study in a rural region in Côte d’Ivoire, a portable digital microscope was compared to a standard of light microscopy and obtained a sensitivity of 85.7% and specificity of 93.3% for *S. haematobium* [10]. Sensitivity may be reduced in light infections and due to day-to-day variation in egg output.

The benefits of the portable digital microscopy technology are multi-fold. First, it may significantly reduce the logistical challenges associated with sample transportation, as each digital microscope weighs less than 1 kg, and image-based diagnosis can be made on-site with the option to transmit images electronically. Second, sample preparation is rapid and simplified, requiring only that 10 ml of urine is filtered through a disposable, mass-produced capillary tube that doubles as the imaging chamber. Third, portable digital microscopy overcomes the need for technical training by allowing personnel to use automated, user-friendly software to capture and transmit images with minimal training. Fourth, artificial intelligence and image processing may automate the diagnosis, further simplifying and accelerating the process [6,14,15]. This can simplify data management and reduce errors due to having this as an automated process. Fifth, given that the same digital microscopes can be reused for thousands of tests, the technology may provide a cost-effective alternative, potentially rapidly expanding the scale of schistosomiasis diagnosis for prevalence surveys in low-resource settings by targeting hard-to-reach communities. Sixth, the microscopy device is battery powered (including the option to be charged by solar power), reducing reliance of constant access to electricity or a generator. Such technology may help meaningfully advance schistosomiasis control and elimination programs, as outlined by the WHO’s recent target product profile [16].

In settings co-endemic for *S. mansoni* and *S. haematobium*, we propose consideration of a mapping strategy that only relies on the collection of urine samples. This strategy would apply urine filtration and portable digital microscopy tools for the diagnosis of *S. haematobium* and POC-CCA assays for *S. mansoni* [17,18], and a strategy with urine-only testing may yield many benefits. This strategy has many advantages over the standard approach of stool microscopy (Kato–Katz) for *S. mansoni* and light microscopy of filtered urine for *S. haematobium*. As an example, a conventional prevalence survey conducted in a suspected endemic area may test ~50 persons per site across 5 sites, for a total survey of 250 people. To evaluate the cost argument for this mapping strategy, a hypothetical cost estimate can be constructed (Table 1). Several factors contribute to the overall cost of a prevalence survey: test kit and supplies, labor, transportation, capital, and overhead costs [19]. Our hypothetical cost estimates focus on test kit and supplies and labor costs, although the mapping strategy will have additional cost savings on transportation (as described below). The supplies, such as the disposable capillary, are assumed to be manufactured at scale. For simplicity, we do not account for the initial capital costs of microscopes, which will be amortized over the number of tests carried out. Capital and overhead costs may not be different compared to a standard survey aside from the portable digital microscopy device.

**Table 1:** Hypothetical and simple cost estimation for a mapping strategy with portable digital microscopy and POC-CCA for *S. mansoni* and *S. haematobium* co-endemic settings.

	*Mapping strategy*	
**Category**	**Portable digital microscopy/POC-CCA** **(proposed)**	**Urine microscopy/Kato–Katz (two samples)** **(status quo)**
*Component costs*		
Test kit and supplies	$2.5 for POC-CCA test, $1 for supplies for portable digital microscopy	$1 per duplicate Kato–Katz with one sample, $1 for supplies for urine microscopy
*Personnel*		
Days of labor	1	2 (due to two stool samples)
Number of technicians per day (per 50 people in a survey)	2 × $70/day	3 × $70/day
**Total cost**		**Total cost**
*Prevalence survey size*		

50 people	$315	$570
250 people	$1,575	$2,850

This is a hypothetical and highly simplified cost estimate meant purely for illustrative purposes on relative cost differences for testing/supplies and labor between mapping strategies; this is not intended for budgeting or a comprehensive cost estimation. These costs focus on the recurring costs of the tests. Cost estimates are based on work in Côte d’Ivoire and may differ substantially between settings and program structure. Transportation, capital equipment, and overhead costs are assumed to be similar between the mapping strategies. We did not account for the cost of the device required for portable digital microscopy or traditional light microscope. The status quo strategy requires 2 days given the need for collection two stool samples on different days. In some cases, one stool sample could also be a reasonable alternative, although would have lower sensitivity and may still require multiple days to complete sample collection and testing.

Abbreviation: POC-CCA, point-of-care circulating cathodic antigen.

There are multiple cost considerations and advantages for a mapping strategy using urine alone and leveraging portable digital microscopy. First, this mapping strategy only uses urine specimens (instead of urine and stool samples), meaning the survey can be much quicker since sample collection can be faster. This may prevent the need for multiple trips to the same site over consecutive days for sample collection (Kato–Katz often relies on multiple stool samples over consecutive days to improve sensitivity; or providing stool sample containers to a region and returning the next day to collect the sample), and the strategy can allow administration of preventive chemotherapy during the same visit if the area is deemed endemic. This may ultimately increase the efficiency of prevalence surveys by allowing for more sites to be visited in a shorter period of time (if the stool-based survey can be completed in a single day that relies on one stool sample, there would be less cost savings, although it would be less sensitive). Second, traditional microscopy requires functional laboratories with laboratory-grade light microscopes, which can cost thousands of dollars. In contrast, portable digital microscopy used in our studies relies on mass-produced mobile phone components and other electronics, which are available at a lower cost compared to traditional microscopy equipment. Third, a major cost driver of using laboratory microscopy is the transportation of samples from site of collection to centralized laboratories (although in some settings field laboratories can be set up). Portable digital microscopy eliminates the need for sample transportation, reducing associated costs. Finally, while an initial investment in portable digital microscopy is required, the overall cost savings over time can be sizable, especially when considering the scalability and potential for community-based screening. The drivers of cost saving include both transition to urine-only testing and use of diagnostic tools that are faster and less reliant on laboratory infrastructure. Urine-only based prevalence survey using POC-CCA and light microscopy would also have cost savings; alternative approaches (e.g., hematuria test strips) exist but have imperfect sensitivity and specificity.

While the proposed strategy is potentially attractive, there are several barriers to its realization that must be overcome. First, further field validation of the accuracy, cost, and implementation challenges of the mapping strategy is needed in a large-scale study. While preliminary data suggests favorable findings, further prospective validation of the sensitivity and specificity of both mobile microscopy and POC-CCA on individuals and community classification accuracy is needed to evaluate this proposed strategy. Second, quality assurance, standardization, and commercial production of portable digital microscopy tools are needed. Most of these tools remain at an “in development” phase; however, implementing these tools at scale would require further commercialization, perhaps requiring investment from funding agencies. Third, since the portable digital microscopy tools have the potential to store personal health information, data security mechanisms to ensure patient privacy need to be implemented [20]. Fourth, the use of POC-CCA removes estimation of infection intensity for *S. mansoni*, which is often a useful indicator of disease burden and aligned with WHO goals [4]. Similarly, POC-CCA does not detect other pathogens (e.g., soil-transmitted helminths, giardia) that might be detected with stool microscopy. Further development of portable digital microscopy for stool samples may offer a quantitative solution in the future. Fifth, while we propose a hypothetical and simple cost estimation for illustrative purposes, further research is needed here. Sixth, to achieve access and scale, future work should ensure commercial access to mobile microscopy devices or availability of open-source 3D printing schematics and manuals on operation. Finally, challenges to adoption of new technologies, technical training, and implementation remain present.

While challenges exist, ongoing advancements in portable digital technology for diagnosis of schistosomiasis, coupled with concerted efforts from the scientific community, funding agencies, and policymakers, can overcome these barriers. The integration of portable digital microscopy into schistosomiasis prevalence surveys holds the promise of improving diagnostic efficiency and advancing the goal of reducing the global burden of this neglected tropical disease.
